# Expert consensus on Prospective Precision Diagnosis and Treatment Strategies for Osteoporotic Fractures

**DOI:** 10.14336/AD.2023.1223

**Published:** 2023-11-26

**Authors:** Yan Hu, Xiaoqun Li, Xiao Chen, Sicheng Wang, Liehu Cao, Hao Zhang, Yunfei Zhang, Zhiwei Wang, Baoqing Yu, Peijian Tong, Qiang Zhou, Feng Niu, Weiguo Yang, Wencai Zhang, Shijie Chen, Qiang Yang, Tao Shen, Peng Zhang, Yong Zhang, Jun Miao, Haodong Lin, Jinwu Wang, Lei Wang, Xin Ma, Hongjian Liu, Ilia Stambler, Long Bai, Han Liu, Yingying Jing, Guohui Liu, Xinglong Wang, Dongliang Wang, Zhongmin Shi, Robert Chunhua Zhao, Jiacan Su

**Affiliations:** ^1^Xinhua Hospital, School of Medicine, Shanghai Jiao Tong University, Shanghai, China.; ^2^First Affiliated Hospital of Naval Medical University, Shanghai, China.; ^3^Shanghai Zhongye Hospital, Shanghai, China.; ^4^Luodian Hospital, Baoshan District, Shanghai, China.; ^5^Tangdu Hospital Air Force Medical University, Xi’an, China.; ^6^Eastern Hepatobiliary Surgery Hospital, Shanghai, China.; ^7^Shanghai Pudong New Area People’s Hospital, Shanghai, China.; ^8^Zhejiang Provincial Hospital of Chinese Medicine, Hangzhou, China.; ^9^Third Affiliated Hospital of Chongqing Medical University, Chongqing, China.; ^10^First Bethune Hospital of Jilin University, Changchun, China.; ^11^HKU Li Ka Shing Faculty of Medicine, Hongkong, China.; ^12^First Affiliated Hospital of Jinan University, Guangzhou, China.; ^13^Third Xiangya Hospital of Central South University, Changsha, China.; ^14^Tianjin Hospital, Tianjin, China.; ^15^Shengjing Hospital of Chinese Medical University, Shenyang, China.; ^16^Shandong Provincial Hospital Affiliated to Shandong First Medical University, Jinan, China.; ^17^Shanghai General Hospital, Shanghai, China.; ^18^Shanghai Ninth People's Hospital, Shanghai Jiao Tong University School of Medicine, Shanghai, China.; ^19^Ruijin Hospital Shanghai Jiao Tong University School of Medicine, Shanghai, China.; ^20^Sixth People's Hospital Affiliated to Shanghai Jiao Tong University, Shanghai, China.; ^21^First Affiliated Hospital of Zhengzhou University, Zhengzhou, China.; ^22^Department of Science, Technology and Society, Bar Ilan University, Ramat Gan, Israel.; ^23^International Society on Aging and Disease, Bryan, TX, USA.; ^24^Institute of Translational Medicine, Shanghai University, Shanghai, China.; ^25^Union Hospital of Tongji Medical College, Huazhong University of Science and Technology, Wuhan, China.; ^26^Department of Pharmacology & Toxicology, University of Arizona, Tucson, USA.; ^27^Institute of Basic Medical Sciences, Chinese Academy of Medical Sciences, School of Basic Medicine, Peking Union Medical College, Beijing, China.

**Keywords:** Osteoporotic fractures, Prospective, Diagnosis and Treatment Strategies, Experts consensus

## Abstract

Osteoporotic fractures are the most severe complications of osteoporosis, characterized by poor bone quality, difficult realignment and fixation, slow fracture healing, and a high risk of recurrence. Clinically managing these fractures is relatively challenging, and in the context of rapid aging, they pose significant social hazards. The rapid advancement of disciplines such as biophysics and biochemistry brings new opportunities for future medical diagnosis and treatment. However, there has been limited attention to precision diagnosis and treatment strategies for osteoporotic fractures both domestically and internationally. In response to this, the Chinese Medical Association Orthopaedic Branch Youth Osteoporosis Group, Chinese Geriatrics Society Geriatric Orthopaedics Committee, Chinese Medical Doctor Association Orthopaedic Physicians Branch Youth Committee Osteoporosis Group, and Shanghai Association of Integrated Traditional Chinese and Western Medicine Osteoporosis Professional Committee have collaborated to develop this consensus. It aims to elucidate emerging technologies that may play a pivotal role in both diagnosis and treatment, advocating for clinicians to embrace interdisciplinary approaches and incorporate these new technologies into their practice. Ultimately, the goal is to improve the prognosis and quality of life for elderly patients with osteoporotic fractures.

Osteoporosis and osteoporotic fractures are major social problems that pose a serious threat to the mobility and health of the elderly population, even in developed countries. The mortality rate of hip fracture patients within 12 months after surgery is as high as 25%; moreover, less than 50% of patients can recover to pre-surgery mobility levels. A clinical study containing 1,151 female patients with hip fractures and 842 with vertebral fractures [1] found that the mortality rates in the first year were 3.8% and 3.1%, respectively, and 33.2% of patients lost the ability to care for themselves. The 2013 survey report from the International Osteoporosis Foundation showed that the average treatment cost for osteoporosis patients with hip fractures in China ranged from $3,600 to $5,000 [2]. Epidemiological research on the burden of osteoporosis-related fractures suggests that the annual incidence of hip fractures in China will rise from 411,000 cases in 2015 to 1 million in 2050; and the healthcare costs of all osteoporosis-related fractures were: $11 billion in 2015, $20 billion in 2035, and $25 billion in 2050 [3].

Distinct from conventional fracture patients, osteoporotic fractures are characterized by poor bone quality, difficulties in reduction and fixation, delayed healing, and a higher risk of re-fracture, making clinical intervention relatively complex. In light of these challenges, the Youth Osteoporosis Study Group of the Orthopedic Branch of the Chinese Medical Association, along with the Youth Osteoporosis Study Group of the Orthopedic Physician Branch of the Chinese Medical Doctor Association, assembled relevant experts to summarize clinical experiences. They proposed a "three-in-one" treatment strategy for osteoporotic fractures [4], emphasizing the concept of rapid bone repair for osteoporotic fractures. Therefore, the core issue in treating osteoporotic fractures lies in rapid healing post-fracture, breaking the vicious cycle of fracture-immobilization-rapid bone loss-delayed healing. The rapidly advancing scientific and technological means have provided new opportunities for the swift healing of osteoporotic fractures. To achieve precise diagnosis and rapid repair of osteoporotic fractures, the expert panel formulated this consensus based on clinical diagnostic and therapeutic needs and relevant technical literature. It outlines the future technologies for precise diagnosis of osteoporotic fractures, potential interventions, and preventive measures, advocating for clinicians to understand, employ, and develop new interdisciplinary techniques, further improving the clinical diagnostic and therapeutic effects of osteoporotic fractures.

## 2. Strategies for future precision diagnosis

Osteoporosis has become a global public health problem, and its complication, fragility fracture, can be disabling and seriously affect patients' quality of life. Early diagnosis of osteoporosis is the key to its treatment and prevention of osteoporotic fractures. Patients with osteoporosis have a reduction in the amount of normally calcified tissue per unit volume of bone, including the organic components of bone and calcium salts, but the ratio of organic components of bone to calcium salt content remains normal [5-7]. Histological changes are thinning of the bone cortex, enlargement of the Haar's canal and reduction of trabeculae, impaired bone mass and reduced bone strength, leading to increased bone fragility and susceptibility to fracture [8-10]. The development of nano-chip and molecular diagnostic technology provides a solid theoretical foundation for the future accurate diagnosis of osteoporosis, while the development and improvement of various imaging technologies provide an effective platform for the diagnosis of osteoporotic fracture.

**Consensus Opinion 1**: Early and accurate diagnosis is important for the diagnosis and treatment of osteoporotic fractures, and nanotechnology can help to rapidly improve the efficiency of sample detection.

Early and accurate diagnosis is important for the diagnosis and treatment of osteoporosis fractures, and osteoporosis patients are often delayed due to untimely early assessment. Diagnostic sensitivity is a key factor in early diagnosis, and the sensitivity and specificity of detection can be significantly enhanced by utilising the unique optical, electrical, thermal, magnetic, and mechanical properties of nanoparticles.

The new nanodiagnostics is the use of nanotechnology in biomedical diagnostics, and protein nano-biochips are devices that are nanotechnology-based biochips and microarrays, a technology that is now beginning to be used in a wide variety of fields [11,12]. The new nanodiagnostics is the use of nanotechnology in biomedical diagnostics. Protein nano-biochips are nanotechnology-based biochips and microarrays of devices that are now beginning to be used in a wide variety of fields. In the field, cDNA can be applied to a slide and passed through a 3D polymer membrane "nano-mesh" on the protein nanochip, termed "Nanonets," facilitating the transformation into target proteins. These methods offer advantages in terms of convenience and rapid results, making them suitable for osteoporotic fracture nanodiagnostics. In the future, nanotechnology might serve as an alternative to conventional approaches for detecting new biomarkers. Nanoparticles have a large surface area and specific physicochemical properties that help identify biomarkers. The structure and shape of the biomarker can also be adapted to bind and chelate the biomarker, which can then be further characterised [13]. Nanodiagnostics, particularly the use of protein nano-biochips and polymer-coated nanoparticles, presents a cutting-edge approach for early and efficient diagnosis of osteoporosis fractures. This technology offers rapid results and the potential to revolutionize biomarker detection in the field, paving the way for improved osteoporosis management.

**Figure 1.**
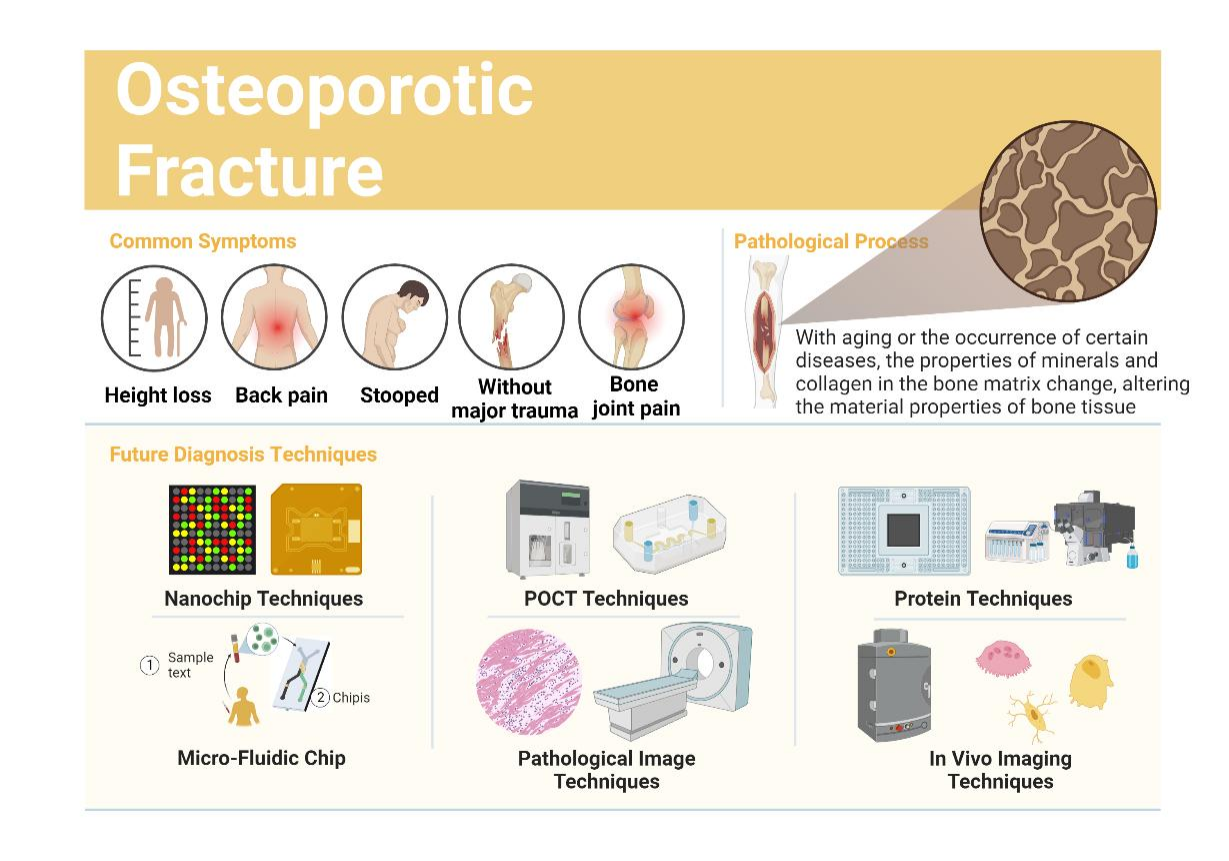
**The potential Future of Osteoporosis diagnostic techniques**. The nanochip and micro-fluidic chip techniques are used for self-diagnosis. And the POCT, pathological image, protein and in vivo imaging techniques provide more accurate diagnostic tools.

**Consensus Opinion 2**: Molecular diagnostic techniques incorporating the latest biomarkers can achieve accurate diagnosis of osteoporotic fracture by simultaneous detection and analysis of multiple biomarkers of osteoporotic fracture.

Molecular diagnostic technology is one of the important frontiers of contemporary medical development, and has gradually gained clinical popularity for its rapid, accurate and efficient performance [14]. Molecular diagnostic technology is one of the important frontier fields of contemporary medical development, and has gradually gained clinical favour with its rapid, precise and efficient performance. Among them, the nucleic acid amplification technology based on polymerase chain reaction (PCR) has become the most popular molecular diagnostic method at present because of its high sensitivity, high specificity, relative simplicity of operation and low cost. However, conventional nucleic acid testing still has certain limitations in time, space and staffing. With the escalating clinical requirements for diagnostic speed and convenience, it has become an urgent need to effectively shorten the waiting time and treatment time.

As a result, through technological innovation and optimisation, the molecular diagnostic technology is fully automated, integrating design to meet the requirements of immediate detection POCT. Molecular diagnostic Point-of-Care Testing (POCT) systems came into being by eliminating the requirements of gene amplification laboratory partitioning and large-scale instrumentation testing steps, to simplify the cumbersome process of data processing, to achieve the testing efficiency of "1+1>2" with the performance of rapidity, accuracy and high efficiency [15]. In recent years, molecular diagnostic POCT has been widely valued, and related new products continue to emerge, presenting a rapid development trend of expanding the scope of application and technological updating and pragmatisation to promote each other, providing stronger technical support for clinical diagnosis and treatment.

**Figure 2.**
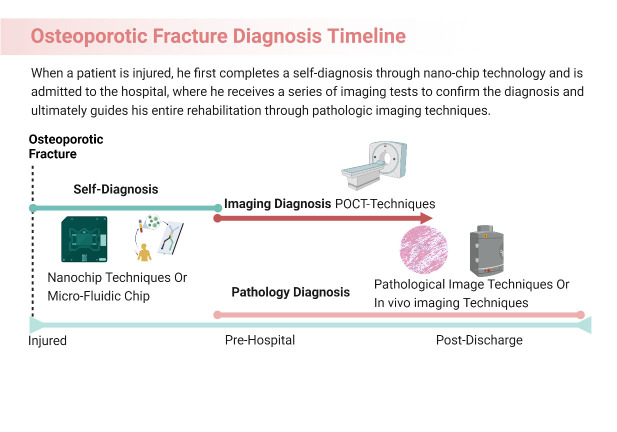
The diagnosis timeline of a patient suffering from osteoporotic fracture in the future.

Molecular diagnostic testing products enable simultaneous detection of multiple markers on a single protein chip. These products can extend their applications to nucleic acid sequencing and protein analysis. Detection tools are becoming smaller and more accessible, including the use of test cards for quantitative analysis, Bluetooth or wireless data transmission, and mobile phone-based testing. At present, POCT products can be applied to medical institutions, in addition to the ones more focused on clinics, medical emergencies and households, so basically a "single copy" is the main mode, and a single test time is relatively long. In Europe and the United States, for example, the market share of products, equipment, lightweight and compact, and a wide coverage of test items, are applicable to a single person osteoporosis fracture biomarker determination and in the future may be widely used.

**Consensus Opinion 3**: One-stop testing of patient serum using microfluidic chips has potential for further development in early emergency care and pre-hospital diagnosis of patients with osteoporotic fractures.

In recent years, the rise and integration of various new technologies and methods have promoted the development, application and renewal of in vitro diagnostic instruments and reagents. In the nano-technology revolution of the 1980s, microfluidic chips were still only a small branch of it. In the late 1990s, after the development of material science and fluid movement technology of microchannels in the study of chip substrate, microfluidic technology also made great progress, finally finding a breakthrough in the application of in vitro diagnostics, re-emerging in the public field of vision, and finally achieving successful commercialisation.

Microfluidic chip [16,17] can integrate a series of basic operation units involved in the fields of chemistry and biology, such as sample preparation, reaction, separation, detection, etc., into a micron-sized chip, and at the same time, the network formed by microchannels, which can run through the whole system, has the advantages of portability, low energy consumption, easy to make, easy to master, etc., in order to make maximum use of the liquids and the surface of the object related to, including laminar flow effect. The system offers portability, low energy consumption, ease of fabrication, and user-friendliness. It harnesses the unique properties associated with liquid and object surfaces, including the laminar flow effect, capillary effect, rapid heat conduction, and diffusion effect. This enables the execution of a comprehensive set of experimental processes on a single chip, including sample inletting, pre-processing, molecular biology reactions, and detection. It is easy to meet the clinical demand for low-dose, more efficient, highly sensitive and rapid separation and analysis of blood from osteoporosis fracture patients. Microfluidic technology, evolving from a small branch of nanotechnology to a successful commercial application, offers a powerful solution for in vitro diagnostics. Its integration of various functions into a compact chip, along with the advantages of portability and low energy consumption, makes it particularly relevant for addressing clinical demands related to osteoporosis fracture patients.

**Consensus Opinion 4**: Diagnostic pathology imaging technology, which allows non-invasive methods of obtaining pathological imaging data from patients, is an important diagnostic basis for the future treatment of osteoporotic fracture.

Doctors rely more and more on medical imaging and pathology to diagnose diseases, and imaging technology has greatly improved doctors' understanding of diseases and diagnosis accuracy. For the diagnosis of osteoporosis fracture, at present, pure imaging technology can not explain the degree of osteoporosis and changes in bone metabolism, which makes pathological technology particularly important. Pathological imaging diagnostic technology, through high-performance imaging equipment, such as computerized tomography (CT), magnetic resonance imaging (MRI), or ultrasound, after the discovery of osteoporosis fracture site, is able to target the specific fracture site, unlimited magnification, magnified to the cellular level, and make a diagnosis of the degree of osteoporosis fracture. Pathology imaging technology involves the visualization and analysis of tissues and cells to understand the structural and functional changes associated with diseases. This technology has evolved significantly, driven by advancements in imaging modalities, computational methods, and molecular techniques. Based on pathology imaging technology, a diagnosis of the degree of osteoporotic fracture is made.

Diagnostic pathology has its own unique advantages. Firstly, the use of diagnostic pathology imaging technology does not require the acquisition of tissue from the patient's body. It can directly magnify, observe, and diagnose an abnormal lesion in the human body [18]. Complications caused by invasive tests or failure to make a full diagnosis due to to small extraction are avoided. At the same time, the diagnostic process is faster and timelier. Traditional pathology examination requires the clinician to obtain a specimen by surgery or puncture, which requires a preoperative preparation process. After obtaining a specimen, the medical staff in the pathology department processes it before analysis and diagnosis can take place. This processing delay can impact the treatment of patients with osteoporotic fractures. Conversely, pathology imaging technology allows for quick and prompt diagnostic conclusions. Furthermore, this technology captures cell morphology in its natural state, aligning better with physiology. Currently, traditional specimen processing may not enable timely treatment. Additionally, after formalin fixation, staining, and other treatments, some intracellular structures may be dissolved or damaged to some extent, potentially affecting the accuracy of the diagnosis. The pathology image diagnostic technologies designed to observe and analyse the lesions in the human body, the cellular morphology and internal structure, blood supply, and the relationship with the surroundings, are in the daily state of existence, providing more objective authenticity. Fourth, the application of technology has no dead zone. Obtaining patient specimens can be challenging for certain diseases. In addition to surgical procedures, factors like patient concerns about pain, reluctance to cooperate, or risk avoidance can hinder specimen collection. Moreover, when lesions are in unusual or hard-to-reach areas, attempts at specimen extraction via puncture may fail, impeding the progress of pathology technology. However, diagnostic pathology imaging technology effectively avoids the above situations, and will not affect the diagnostic pathology technology because of the patient's fear or the special site of the lesion [19]. In conclusion, pathology imaging technology continues to evolve, integrating with other disciplines and technologies to provide a comprehensive understanding of diseases. The future holds promise for more advanced, personalized, and accessible diagnostic and research tools.

**Consensus Opinion 5**: Medical molecular imaging in vivo is an important future technology for visualising and confirming the diagnosis of osteoporotic fractures.

Molecular imaging refers to the application of medical imaging methods to achieve the display of biological and pathological processes at the cellular, molecular or genetic level in vivo, and can further achieve the early quantitative and qualitative diagnosis of diseases related to the study of the frontier disciplines [20,21]. Medical molecular imaging is a field that combines molecular probes and nanomaterials with various imaging methods. This combination allows for non-invasive imaging of specific molecular targets within the body. It's a multidisciplinary field that intersects with molecular biology, nanomaterials, medical imaging, nuclear medicine, and computer science. Within this field, various applications exist, including tumor targeting imaging, gene imaging, receptor imaging, single-cell tracing, and the study of cellular signaling pathways. Medical molecular imaging is an important component of future medicine, contributing to early disease diagnosis and treatment in both the present and the future.

Molecular imaging allows the visualisation of cellular functions and the continuous tracking of molecular delivery without interfering with the metabolic functions of the organism. Molecular imaging has a diverse research and therapeutic potential in the medical field, not only for the early diagnosis of diseases in cancer, neurological and cardiovascular areas, but also for the improvement of conventional treatments for these diseases and the development of novel biomarkers by means of molecular and genetic level. In addition, it can optimise novel drugs for preclinical and clinical trials and detect so-called pre-disease states or molecular states that occur before the typical symptoms of a disease are detected. In recent years, the term "molecular imaging" has been applied to a variety of microscopy and nanomicroscopy techniques, including live-cell microscopy, total internal reflection fluorescence microscopy, stimulated emission loss nanomicroscopy, and atomic force microscopy, and is believed to play an important role in the future treatment of osteoporotic fractures. In recent years, "molecular imaging" has broadened to include microscopy and nanomicroscopy techniques like live-cell microscopy and atomic force microscopy. This expansion is expected to play a crucial role in the future treatment of conditions such as osteoporotic fractures.

**Figure 3.**
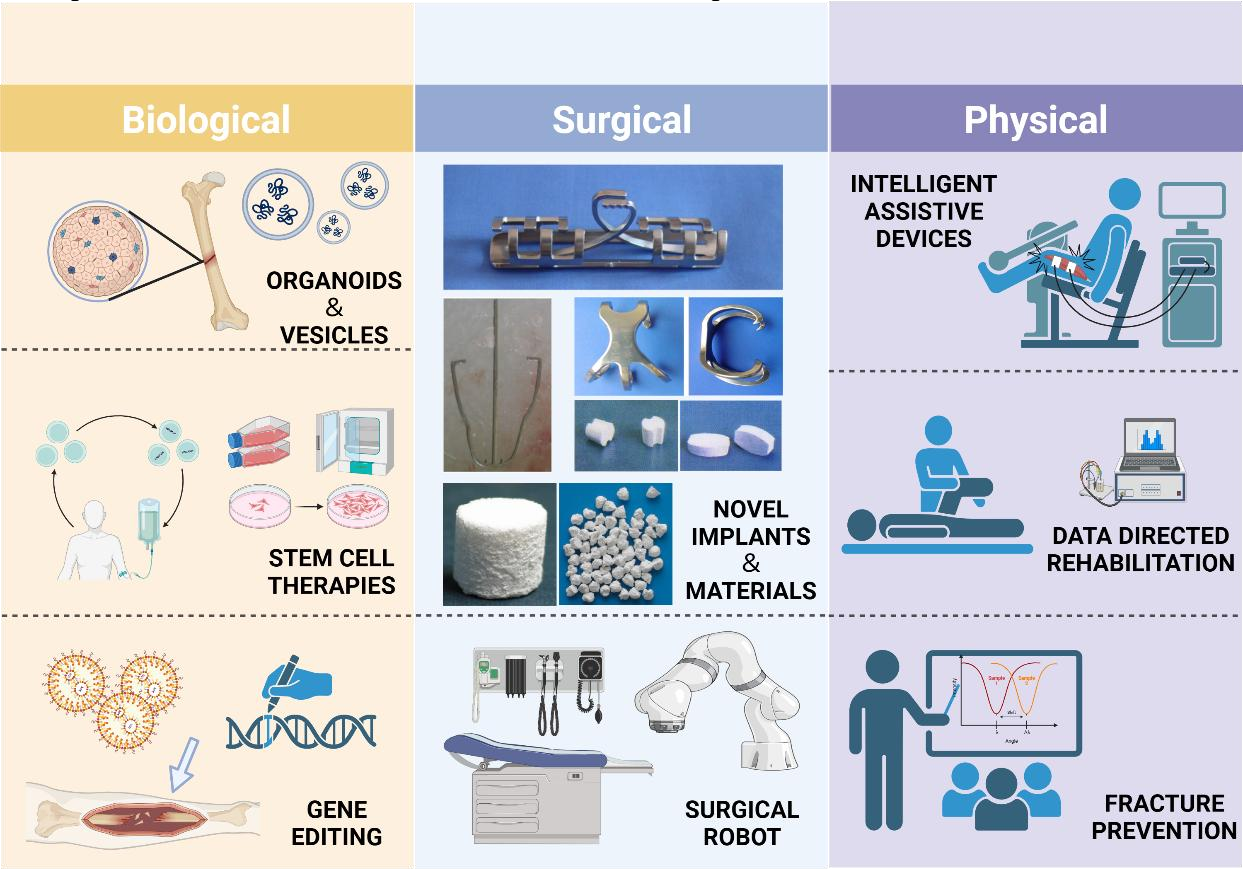
**The future therapies for osteoporotic fractures**. Organoids and vesicles, stem cell therapies and gene editing enable biological therapies. Implants, biomaterials, and surgical robots are well recognized to be potential, as well as big data directed rehabilitation and prevention.

## 3. Strategies for future clinical therapies and prevention

The core challenges in treating osteoporotic fractures lie in poor bone quality, slow fracture healing, high surgical complexity, and elevated risk of re-fracture. Future intervention strategies will focus on four primary aspects: improving local bone metabolism, accelerating fracture healing, precise personalized reduction and fixation, and preventing subsequent fractures. The advancements in intelligent biomaterials and the philosophy of material biology [22], coupling of in-depth research in bone-related organs and their derivatives [23] offer fresh perspectives for enhancing bone metabolism and promoting fracture healing. Artificial intelligence (AI) surgical robots and preoperative planning [24] further aid in surgical reduction and fixation, while the development of big data and patient information cloud platform technologies [25] provides technical support for the prediction and early intervention of osteoporotic fractures.

**Consensus Opinion 6**: Intelligent bone repair biomaterials respond to various biophysical factors during the bone metabolism process and make pre-set reactions. These factors include but are not limited to temperature, pH level, electrical charge, and illumination.

With the continuous advancement of bio-pharmaceutical means, the term "intelligent" is used to describe novel biomaterials that can respond to external stimuli in specific and predictable ways [26].

Intelligent responsive biomaterials can undergo reversible changes in structure or properties in response to external stimuli or environmental conditions, realizing specific biomedical applications [27]. These materials are typically composed of biodegradable polymers, bioactive molecules, and other biological components. Intelligent responsive biomaterials can generate physicochemical changes or release specific bioactive substances depending on different stimuli, such as temperature, pH level, electric fields, and illumination. These materials hold vast potential for various medical applications, such as drug delivery systems, tissue engineering, biosensors, etc.

For instance, a novel CuS nanoparticle-PEG soft hydrogel-coated 3D hard polycaprolactone scaffolds were reported recently [28], when treated with near-infrared light irradiation, drug stored inside the scaffold could be released and efficently regulate local microenvironment. The potential of these intelligent responsive biomaterials lies in their ability to generate physicochemical changes or release specific bioactive substances tailored to different environmental conditions, showcasing their promise in diverse medical applications.

**Consensus Opinion 7**: Organoids and their derivatives, characterized by low immunogenicity and high bioactivity, represent a key development direction in the field of precise bone repair in the future.

Organoids are clusters of cells with partial organ activity and physiological functions, formed by in vitro induction of stem cells [29]. Since the first report of intestinal organoids in 2009 [30], lung [31], kidney [32], liver [33], brain [34] organoids have been sequentially designed and constructed, showing tremendous potential in areas like drug screening and disease modeling. Bone organoids generally utilize bioactive materials as scaffold structures, loaded with various stem cells to form tissues with bone physiological functions. Current research on bone organoids is still in its infancy, and there are many technical difficulties in areas such as regulating tissue structure and forming spatial structures that include cortical bone, cancellous bone, and blood vessels. However, bone organoids have unparalleled advantages in the treatment of osteoporotic fractures: first, bone organoids are sourced from the patient's own stem cells, avoiding the risks of artificial bone rejection and disease transmission; second, a large amount of bioactive factors is continuously produced locally in the organoids during stem cell proliferation and differentiation, offering sustainability compared to the traditional addition of bioactive factors in tissue engineering; third, organoids are living tissues that can regulate local metabolic conditions through cellular derivatives like exosomes, achieving rapid, high-quality bone repair. Similar to body tissues and organs, organoids produce a series of derivatives represented by exosomes or extracellular vesicles during the nutritional metabolism process. These derivatives often carry a plethora of growth factors and cellular communication media, effectively stimulating local bone metabolism and promoting in situ bone regeneration. Moreover, these living tissues continuously produce bioactive factors locally, promoting sustainable and high-quality bone repair. Organoids, similar to natural tissues, generate derivatives like exosomes during metabolic processes, carrying growth factors and communication media that stimulate local bone metabolism and facilitate in situ bone regeneration.

**Consensus Opinion 8**: AI assistance in orthopedic surgical techniques has gradually been implemented, and future AI-assisted surgical planning and intraoperative procedures will benefit the precise reduction and fixation of osteoporotic fractures.

Robot-assisted surgery has become an important application in the medical field. AI-assisted fracture reduction refers to the use of artificial intelligence technology to aid doctors in the process of fracture reduction. Traditional fracture reduction is usually performed manually by experienced physicians. However, artificial intelligence can provide assistance and guidance to help doctors carry out the reduction more accurately. By integrating robotic technology with AI algorithms, physicians can perform more precise, safe, and minimally invasive surgical procedures. Robotic systems can provide a more stable operating environment and enhance the accuracy and success rate of surgery through AI-assisted navigation and real-time feedback. Surgical robots have found some application in joint replacement surgeries [35], although robot-assisted fracture surgeries have not yet been reported on a large scale. Basic techniques like percutaneous locator and man-machine interactive system are well-established and could be directly applied on trauma surgery robots. Especially, machanical arms that handle surgical operations should be the critical part when designing trauma robots. With the accumulation of experience in robot-assisted surgery and the optimization of relevant control technologies, AI-assisted surgical skills will further improve the accuracy and long-term effects of fracture surgeries.

It must be noted that, although AI has made significant progress in the medical field, there are still some challenges faced in its practical application, including issues related to data privacy and security, algorithm interpretability, and clinical validation [36]. Therefore, further research and development will still be needed in the future to ensure the reliability and sustainable development of AI technology in medicine. Despite significant progress, challenges in AI's practical application in medicine, such as data privacy, security, algorithm interpretability, and clinical validation, still exist. Ongoing research and development are crucial to ensuring the reliability and sustainable development of AI technology in the medical field.

**Consensus Opinion 9**: Big data platforms can integrate and analyze clinical data to predict disease risk and provide guidance for the prevention of recurrent fractures and health management.

Big data platforms can consolidate and analyze large-scale medical data, bioinformatics data, environmental data, and more, for monitoring disease transmission and epidemiological trends [37]. Through data analysis and modeling, they can forecast the occurrence and transmission risks of diseases, offering early warnings and intervention measures. In the field of osteoporosis and osteoporotic fractures, the big data platforms' role in disease prevention is mainly reflected in four aspects:

### 1)Disease risk assessment

Big data platforms can integrate and analyze extensive medical data, genomics data, environmental data, and other data sources to help predict individual risks of bone loss and fractures. By creating predictive models and algorithms, individualized fracture risk assessments can be provided based on personal movement characteristics and behavioral patterns [38], thereby guiding individual health management and the formulation of preventive measures.

### 2)

Epidemiological surveillance: By collecting and analyzing data from various sources, including social media data, search engine query data, disease reporting data, and more, big data platforms can track and monitor the incidence and trends of osteoporosis and osteoporotic fractures. This enables health departments and relevant agencies to respond earlier to the trends in osteoporosis and osteoporotic fractures under the context of an aging population and take corresponding measures for control and prevention.

### 3)Health behavior analysis

The big data platforms for osteoporotic fractures can collect personal health and activity data, such as physical activity, dietary habits, sleep quality, and living environments. By integrating this with other environmental and social data, the platforms can analyze and assess an individual's health behaviors and lifestyle. Using these analytical results, individuals can be educated about their health conditions and provided with personalized lifestyle recommendations and exercise guidelines, promoting changes in health behavior and the prevention of fractures.

**Figure 4.**
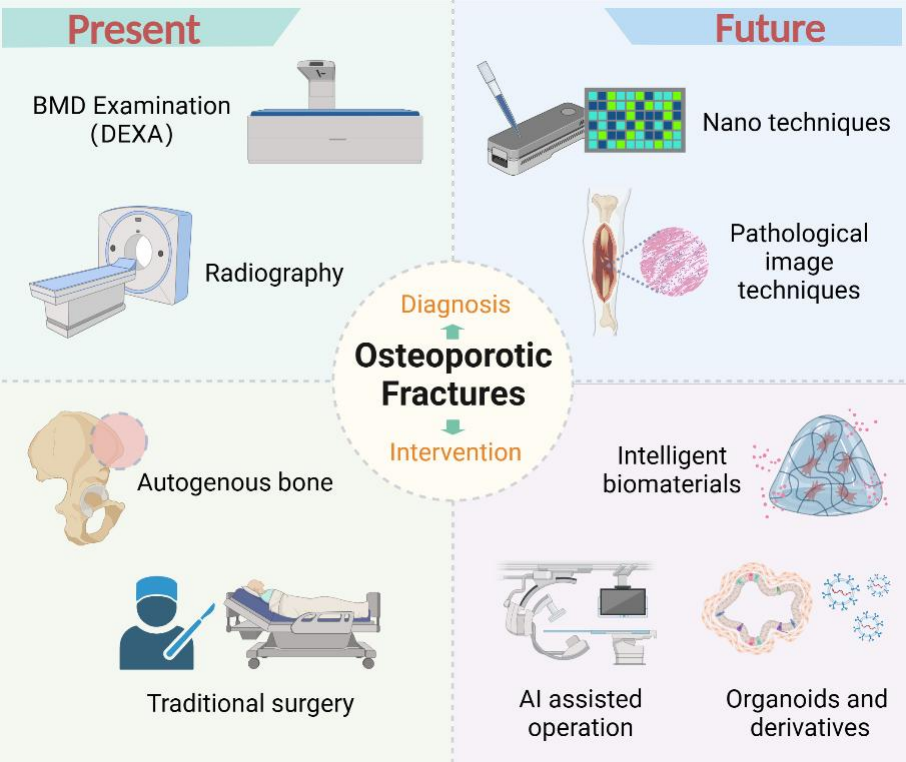
**The comparison between the diagnosis and intervention of osteoporotic fractures at present and in the future**. We use DEXA and manual surgeries at present, but might launch nano techniques and other fancy technologies in years to come.

### 4)Data-driven public health policy

The big data platforms for osteoporotic fractures can offer extensive data support for the formulation and evaluation of public health policies. By analyzing population health statuses, socio-economic data, and environmental data, potential health risk factors can be identified. This information can guide the development of targeted policies and intervention measures. For instance, if a certain residence area shows a fracture incidence rate notably higher than the norm, it might be advisable for relevant departments to consider improvements, such as enhancing community lighting or addressing nutritional statuses.

It's essential to note that when utilizing big data platforms for disease prevention, data privacy and security are significant considerations [39]. There must be assurances in place for data anonymization and protection, and adherence to relevant legal regulations and ethical guidelines must be observed. There are several tips to avoid ethical problems during AI-enabled clinical research, including data anonymization and de-identification, data encryption, access control, informed consent, transparency and communication and data governance and auditing.

## 4. Conclusion and Outlook

The rapid advancement of scientific and technological innovation offers both novel opportunities and challenges to medical science (Fig. 4). Imaging technology and artificial intelligence present opportunities for medical diagnosis and treatment decisions, while gene editing and big data collection technology likewise pose ethical and privacy challenges. As clinical practitioners embrace new technologies, they must also maintain a firm stance on safeguarding patient interests and upholding the fundamental principles of human ethics.

With the ongoing deterioration of population demographics, the risk and harm of osteoporotic fractures are steadily rising. Future strategies for diagnosing and treating osteoporotic fractures will focus primarily on precise diagnosis and rapid healing. This consensus emphasizes new technologies that may play a key role in the precise diagnosis and treatment of osteoporotic fractures in the future, guiding clinicians' sensitivity and attention to new technologies and concepts without recommending them as actual treatment plans. As the understanding of the disease and medical technology continue to grow and evolve, some contents of this consensus will be further updated and refined.
